# Brain-movement relationship during upper-limb functional movements in chronic post-stroke patients

**DOI:** 10.1186/s12984-024-01461-3

**Published:** 2024-10-22

**Authors:** C. O. Muller, G. Faity, M. Muthalib, S. Perrey, G. Dray, B. Xu, J. Froger, D. Mottet, I. Laffont, M. Delorme, K. Bakhti

**Affiliations:** 1 EuroMov Digital Health in Motion, Univ Montpellier, IMT Mines Ales, Montpellier, France; 2 Physical Rehabilitation and Medicine, CHU Montpellier, Montpellier, France; 3 Physical Rehabilitation and Medicine, CHU Nîmes, Le Grau du Roi, Nîmes, France; 4 Silverline Research, Oxford, United Kingdom

**Keywords:** Neuroplasticity, Sensorimotor cortex, fNIRS, fEEG, Reaching and tracing tasks, Stroke, Upper limb

## Abstract

**Background:**

Following a stroke, brain activation reorganisation, movement compensatory strategies, motor performance and their evolution through rehabilitation are matters of importance for clinicians. Two non-invasive neuroimaging methods allow for recording task-related brain activation: functional near-infrared spectroscopy (fNIRS) and electroencephalography (fEEG), respectively based on hemodynamic response and neuronal electrical activity. Their simultaneous measurement during movements could allow a better spatiotemporal mapping of brain activation, and when associated to kinematic parameters could unveil underlying mechanisms of functional upper limb (UL) recovery. This study aims to depict the motor cortical activity patterns using combined fNIRS-fEEG and their relationship to motor performance and strategies during UL functional tasks in chronic post-stroke patients.

**Methods:**

Twenty-one healthy old adults and 21 chronic post-stroke patients were recruited and completed two standardised functional tasks of the UL: a paced-reaching task where they had to reach a target in front of them and a circular steering task where they had to displace a target using a hand-held stylus, as fast as possible inside a circular track projected on a computer screen. The activity of the bilateral motor cortices and motor performance were recorded simultaneously utilizing a fNIRS-fEEG and kinematics platform.

**Results and conclusions:**

Kinematic analysis revealed that post-stroke patients performed worse in the circular steering task and used more trunk compensation in both tasks. Brain analysis of bilateral motor cortices revealed that stroke individuals over-activated during the paretic UL reaching task, which was associated with more trunk usage and a higher level of impairment (clinical scores). This work opens up avenues for using such combined methods to better track and understand brain-movement evolution through stroke rehabilitation.

**Supplementary Information:**

The online version contains supplementary material available at 10.1186/s12984-024-01461-3.

## Background

Due to its prevalence, functional non-recovery of the paretic upper limb (UL) is a critical concern in stroke rehabilitation [1]. UL functional recovery is mainly attributed to plastic reorganization within the human brain [2, 3], and post-stroke patients often demonstrate abnormal brain activation in comparison to healthy individuals. When using the paretic hand, patients with stroke show increased contralesional and ipsilesional sensorimotor network activation compared to healthy individuals [4], as well as increased activations of contralesional primary motor cortex and bilateral premotor and supplementary motor areas [5]. During the process of functional paretic arm recovery, there is a progressive evolution towards a more “normal” lateralization of the primary sensorimotor cortex [6–10], which underlines the potential of monitoring brain reorganization to predict patients’ responses to rehabilitation [11]. Brain reorganization is classically assessed by functional magnetic resonance imaging (fMRI), mostly in the supine position and during moderately functional tasks such as thumb-finger opposition or elbow flexion-extension [12]. To monitor brain activations under more ecological conditions, i.e., during upright, unrestrained, functional tasks, it is possible to use portable brain imagery techniques such as functional near infrared spectroscopy (fNIRS) and functional electroencephalography (fEEG).

The fNIRS method detects variations in blood-oxygen level-dependant response, as in fMRI [13], and can do so under more ecological conditions [14]. FNIRS measures both oxygenated (HbO_2_) and deoxygenated (HbR) hemoglobin in the cerebral cortex blood vessels, and has been previously used to measure sensorimotor network activation during UL movements in healthy young adults [15, 16], older healthy adults [16, 17] and stroke patients [18, 19]. In fully UL functional tasks, such as reaching, studies have identified a bilateral sensorimotor cortex (SM1) activation pattern [16, 20]. Nevertheless, to the best of our knowledge, only one recent study investigated SM1 activation in a stroke population using fNIRS during a reaching task under ecological conditions [18]. They found enhanced ipsi/contralesional SM1 activation in the stroke patients despite poorer motor performance in reaching and grasping.

The fEEG method detects direct variations in electrical currents at the scalp due to local electric fields produced by neuronal activity [21]. Event-related power changes within specific frequency bands (alpha-mu – 8 to 13 Hz and beta – 14 to 29 Hz) reflect the balance between excitation and inhibition in the sensorimotor network [22], classically with an event-related desynchronization (ERD, i.e. power decrease) at movement execution and an event-related synchronization (ERS, i.e. power increase) at rest [23]. In patients with stroke, a number of studies have shown a relationship between the magnitude of the ERD in the lesioned hemisphere and the paretic UL function [24–26].

Coupling fNIRS and fEEG could provide a better spatio-temporal view of SM1 brain activation patterns in both hemispheres [27]. However, to better understand SM1 activity during fully functional UL tasks, it is important to complement functional brain imaging with kinematic assessments [16]. During forward-reaching tasks, stroke patients often exhibit non-mandatory trunk compensation, i.e. even if they can do with their paretic UL alone, they favour trunk flexion to the detriment of arm use [28, 29]. Unfortunately, this non-use of the paretic UL [30] can lead to maladaptive brain plasticity [31] and hinder functional recovery [32]. Overall, it is now clear that non-mandatory trunk compensation and associated non-use have an impact on the plastic reorganisation of the brain (for a review, see [33]). Thus, investigating how trunk compensation affects SM1 activations during different functional UL tasks (detailed description of UL tasks in Sect. “Experimental design”) may help to understand the mechanisms underlying functional recovery [18].

The primary aim of the present study was to investigate bilateral SM1 activation during functional UL tasks in people with and without stroke. We hypothesised increased SM1 activation in the stroke cohort, both in the ipsilesional and contralesional hemispheres and particularly during performance of the paretic UL. Additionally, we investigated the effect of stroke on the relationship between brain activation patterns and motor performance. Our hypothesis was that individuals in the stroke group would perform worse when using their paretic arm, and that SM1 activation in the injured hemisphere would be positively correlated with task performance.

## Materials and methods

### Participants

The study cohort consisted of 21 post-stroke patients and 21 healthy adults. For the stroke group, the inclusion criteria were to: (i) be aged between 18 and 90 years old, (ii) be at more than 3 months of a first cerebrovascular accident of any aetiology (hemorrhagic or ischemic; participants with several strokes were excluded), and (iii) have an UL motor impairment with FM-UE ≥ 15 [34]. The non-inclusion criteria were to: (i) have hemineglect or severe attentional problems (omission of more than 15 bells on the Bell’s test; [35], (ii) have aphasia of comprehension dysfunction (Boston Diagnostic Aphasia Examination < 4/5; [36], and (iii) have severe cognitive dysfunction (Mini Mental State Examination-MMSE < 24; [37]. To be included, the healthy adults had to be aged between 60 and 90 years old (to fit with the stroke group age) and to be right-handed assessed by the Edinburgh Handedness Inventory [38]. Exclusion criteria were the existence of neurological (including a history of traumatic brain injury) or motor disorders at the level of the upper limb (history of tendinous disease, arthritis, surgery). Healthy participants were recruited via local association, while stroke ones were recruited at the beginning of a rehabilitation protocol (ReArm project, Clinical trial identifier: NCT04291573, 2nd March 2020).

Table 1 provides detailed participant information, including gender, age, lesioned side, laterality, and clinical scores (refer to the clinical assessments section for additional details). For the stroke group, Table 2 presents all patients’ demographic data and clinical history. At the time of the experiment, patients were not included in any intensive acute rehabilitation, and were just following maintenance therapy depending on their needs.

In accordance with the Declaration of Helsinki, this study was approved from the French Research Ethics Committee, (Comité de Protection des Personnes-CPP SUD-EST II, N°ID-RCB: 2019-A00506-51, http://www.cppsudest2.fr/) for the stroke patients, and from the local Ethics Committee of the EuroMov DHM laboratory for the healthy subjects (EuroMov IRB, number 1912B). All participants provided informed written consent prior participation in the study.

**Table 1:** Characteristics of the participants for each group (*n* = 21)

Characteristics	Healthy group	Stroke group
Age (years) (SD)	73.1 (± 6.7)	64.4 (± 10.2)
Sex (female/male)	11/10	6/15
Handedness score (SD)	0.96 (± 0.08)	-
Paretic arm (right/left)	-	8/13
FM-UE	-	48.7 (± 5.9)
WMFT	-	57.3 (± 9.8)
BBT ratio	-	54.0 (± 25.1)

BBT ratio = (paretic score / non-paretic score) * 100. Group comparison showed a significant difference in age (T-test, *p* = .002) and a non-significant difference in sex (Chi-square, *p* = .116)

**Table 2:** Demographic information, clinical data and lesion information

*P*	Age	Sex	Hemisphere lesioned	Months since stroke	HD before stroke	Paretic arm	Lesion localisation	Vascular territory	Type of stroke	Sensitivity deficit	Spasticity	MMSE	FM-UE	BI
1	62	M	L	8	R	R	subcortical	Brainstem	Is	yes	no	27	45	85
2	61	M	R	73	R	L	cortical	MCA	Is	yes	no	30	55	95
3	52	M	R	84	R	L	cortical	MCA	Is	yes	yes	24	51	90
4	63	M	L	88	L	R	cortical	MCA, ACA	Is	yes	no	28	44	95
5	70	M	R	98	R	L	cortical	MCA	Is	yes	no	29	51	100
6	73	F	R	223	R	L	cortical	ACoA	H	no	no	27	53	95
7	63	F	R	21	R	L	cortical	-	Is	no	yes	28	27	90
8	57	F	R	10	R	L	cortical	PO lobe	H	no	no	25	60	90
9	74	M	R	49	R	L	cortical	MCA	Is	no	no	28	50	85
10	37	M	R	32	L	L	subcortical	MCA, ACha	Is	no	yes	29	46	95
11	68	M	R	43	R	L	cortical	MCA	Is	yes	no	24	47	95
12	76	M	L	93	R	R	subcortical	None (H stroke)	H	yes	no	29	41	90
13	62	F	R	12	R	L	cortical	MCA	Is	yes	no	28	45	85
14	49	F	R	25	R	L	cortical	MCA	Is	-	yes	27	54	95
15	82	M	L	45	R	R	-	-	Is	yes	-	29	58	100
16	72	M	L	4	R	R	subcortical	ACha	Is	no	no	29	44	90
17	66	M	L	9	R	R	subcortical	ACha	Is	no	no	30	38	95
18	73	M	L	9	R	R	subcortical	brainstem	Is	no	yes	30	36	25
19	71	F	L	18	R	R	subcortical	None (H stroke)	H	yes	no	25	57	95
20	62	M	R	3	R	L	subcortical	None (H stroke)	H	yes	no	29	46	90
21	60	M	R	9	R	L	subcortical	MCA	Is	no	no	27	43	90

*Abbreviations* M, male; F, female; R, right; L, left; HD, hand-dominance; MCA, middle cerebral artery; ACoA, anterior communicating artery; ACA, anterior cerebral artery; ACha, anterior choroidal artery; PO, parieto-occipital; Is, Ischemic; H, Hemorrhagic; MMSE, mini mental state evaluation (score/30); FM, Upper Limb Fugl-Meyer (score/66); BI, Barthel index (score/100). The severity of the motor impairment was evaluating using the FM-UE in accordance with the motor impairment classification in clinical and research settings [39]

### Experimental design

Each participant engaged in an hour-long session in a quiet isolated room. The participants were equipped with the fNIRS-fEEG neuroimaging systems and performed two functional UL tasks while seated: a paced reaching arm task and a circular steering task. The setup permitted synchronized recording of UL kinematics and SM1 activity (fNIRS and fEEG) using lab streaming layer (LSL, https://github.com/labstreaminglayer/App-LabRecorder). More comprehensive details about the functional motor task methodology can be found in our recent methodological paper (see Fig. 5 in [40]).

### Upper-limb function assessments

All participants performed the two functional UL tasks, as detailed in earlier studies [16, 40]. We chose and developed the functional proximal UL tasks that could provide relevant kinematics parameters to understand the movement reorganization (i.e., trunk compensation, movement time, accuracy, speed, performance [28, 29, 41]). There was a gap in the literature at this level, as most of the research projects on task-related brain activity were focused on for distal tasks or tasks that did not used the entire UL (i.e., from trunk to wrist). Since full mobility of the UL is necessary in everyday life activities, it was applicable to use standardized UL movement tasks such as reaching tasks and circular trajectory tracking tasks [16, 42]. Moreover, in the context of stroke, proximal movements, such as arm reaching, can be used to assess patients at the beginning of the recovery process and patient with greater impairment. Indeed, since the process functional recovery has been shown to be proximo-distal direction, patients are most likely to first recover at the level of the proximal UL movements. The reaching task, with maximal condition (maximal arm use with trunk restrained) and spontaneous condition (spontaneous arm use), was previously developed to identify trunk compensation [28], and we further designed the task with a paced rhythm (5 movements per 20s) to particularly enable fNIRS recordings of brain activity changes. The addition of the circular steering task was done in order to allow for a proximal UL performance-based task focused on speed rather than accuracy thus allowing brain activity to be extrapolated to performance.

#### Paced reaching task

Participants were seated on a chair fitted with armrests and were instructed to reach a target (a table tennis ball) placed in front of them at a height of 80 cm and a distance which facilitated the complete extension of the arm. A Kinect sensor (V2, Microsoft, USA), sampled at 30 Hz, was positioned 1.70 m above and 1.60 m away from the target. Participants had to reach the target by extending their arm in two conditions: (i) spontaneous condition (i.e., spontaneous arm use, SAU), and (ii) maximal condition (i.e., maximal arm use, MAU), wherein their shoulders were constrained to minimize trunk movements. Each block consisted in five movements per 20-second block, timed to 4s vocal prompts (“go” for 2s; “stop” for 2s) and was interspaced by 20s of rest. After a familiarization block with each arm, participants completed three blocks using their non-dominant/paretic hand, followed by three blocks using their dominant/non-paretic hand in the spontaneous condition. Then, participants repeated the task for three blocks with each hand under the maximal condition.

#### Circular steering task

This task was based on the speed-accuracy trade-off [43]. Participants were seated on a chair in front of a horizontal graphic tablet (A3 size; Wacom, Kazo, Japan) equipped with a stylus affixed to a mouse pad, facing a 24-inch vertical screen projecting a circular target (33-inch circumference) with a 2 cm tunnel. A Kinect was placed above the graphic tablet at the height of 1.70 m. The task was delivered using a lab-made software, the LSL-Mouse (https://github.com/KarimaBak/LSL-Mouse). Participants were instructed to move a cursor as fast as possible in a clockwise direction. During the familiarization phase, participants were instructed to accelerate if movement trajectory errors (any instances outside the 2 cm circular tunnel boundaries) were below 15% (based on pilot testing). The task comprised three blocks for each arm (20s of task with 20s of rest), commencing with their non-dominant/paretic hand.

#### Clinical assessments of paretic upper limb impairment

In conjunction with the functional kinematics and brain evaluation, patients’ UL motor function was appraised through clinical evaluations. We utilized several recognized and validated tests, including the FM-UE [34, 39], the Box and Block test (BBT [44]), the Wolf-motor function test (WMFT [45]), the Barthel Index (BI [46]), and the Proximal-arm non-use test (PANU [28, 29]). Comprehensive details of these evaluations are described in the cited references.

The FM-UE assesses upper limb motor impairment, while the BBT measures arm and hand grasping function. WMFT evaluates upper limb function, and the BI measures overall functional recovery (independent function in activities of daily living). The PANU test quantifies the amount of shoulder and elbow movements that a post-stroke individual does not use spontaneously, but can use when forced to do so. These tests collectively provide a comprehensive overview of the paretic UL’s functional capacity and impairment (for the FM-UE) level in stroke patients.

### Brain activity (fNIRS and fEEG)

Participants wore a custom neoprene head cap equipped with a combined fEEG-fNIRS system to monitor brain activity within the left and right SM1 regions during both functional motor tasks. We utilized a wireless Starstim fNIRS integration system (Starstim8, Neuroelectrics, Barcelona, Spain; Octamon+, Artinis Medical Systems, Elst, The Netherlands) to measure fEEG and fNIRS signals. Details regarding the placement of the 16 channels, comprising 4 fNIRS and 4 fEEG channels per SM1 hemisphere, are outlined in a previous article (see Fig. 1 in [16]).

The 8 fEEG electrodes were positioned in and around the SM1 cortices: C4, FC2, FC6, CP2 in the right hemisphere and C3, FC1, FC3, CP1 in the left hemisphere, in alignment with the international 10–10 system. The electrodes (NG Geltrode, Neuroelectrics, Spain) were filled with electro-gel (Signa Gel®). Using an ear clip, reference electrodes (CMS, DRL) were placed over the right earlobe. The fEEG signals were sampled at a rate of 500 Hz. We controlled the wifi- fEEG device via a software interface (Neuroelectrics Instrument Controller, NIC v 2.0).

For the fNIRS recording, we used a continuous-wave system employing two wavelengths to capture changes in HbO_2_ and HbR overlying the left and right SM1, sampling at 10 Hz. The two receivers were positioned at the C1 and C2 locations of the 10–10 fEEG system, with four transmitters placed 3 cm from the receivers using plastic holders. The fNIRS Bluetooth device was managed through a software interface (Oxysoft, v3.2.51.4, Artinis Medical Systems, Elst, The Netherlands).

Following the equipment setup, participants were asked to perform a wrist extension task to verify if the movement induced a hemodynamic response in the SM1.

### Data analysis

#### Task performance

The paced reaching and circular steering task kinematics analysis was undertaken based on previous work [28, 29, 47] and LSL-Kinect software (LSL-KinectV2: https://github.com/KarimaBak/LSL-KinectV2). For the paced reaching task, we calculated the proximal-arm non-use (%) and the hand mean velocity (mm/s). For both tasks, we calculated as trunk compensation parameter, the range of trunk anterior flexion (°) representing the use of the trunk to realize the reaching movement. And, we calculated, as arm use parameters the range of elbow extension (°) representing the use of whole arm to perform the movement.

We assessed the speed-accuracy trade-off during the circular steering task using the Index of Performance (IP_e_ in bits/s [48]). We calculated the Index of Effective Task Difficulty (ID_e_) with the formula: $\:{ID}_{e}=\:\frac{2\pi\:R}{{W}_{e}}$, where R represents the subject’s mean circular path radius, and W_e_ denotes the effective path width. We determined W_e_ using MacKenzie’s formula [49]: $\:{W}_{e}=\:\sqrt{2\pi\:e}*\sigma\:$, where σ is the standard deviation of the radius. We then computed IP_e_ by dividing ID_e_ by the movement time (MT). In addition, we calculated the speed as laps per second and accuracy as bias (W_e_/W, following [49]) of the movement.

#### Brain activity (fNIRS and fEEG)

We processed all fNIRS raw data using the HOMER toolbox in MATLAB (Homer2 NIRS processing package, [50]) with the files generated by the Lab Recorder (xdf files). Pre- and post-processing steps are detailed in a previous study [16] and a flowchart presenting these steps is available in the supplementary materials (file 1). We used the relative changes (Δ) in peak HbO_2_ concentration as an indicator of brain activity.

We analysed all fEEG data using the EEGLAB toolbox on MATLAB ([51], version 2021.1), with the files generated by the Lab Recorder (xdf files). Details of pre- and post-processing steps are provided in a previous study [16]. We calculated the event-related spectral perturbations (ERSP) in the alpha (8–13 Hz) and beta (14–29 Hz) rhythms, revealing average power changes in these specific time frequencies. This information provides insight into event-related desynchronization (ERD; power decrease in a specific frequency band relative to baseline, i.e., rest) and synchronization (ERS; power increase in a specific frequency band relative to the task). For fEEG and fNIRS analyses, parameters were averaged by tasks (paced reaching; circular steering), hand condition (dominant / non-paretic; non-dominant / paretic), and hemisphere (contralateral / ipsilesional; ipsilateral / contralesional).

### Statistical analyses

Statistical analyses were performed using R software (version 4.2.1) and the ggplot2 [52], dplyr [53] and rstatix [54] packages. Parametric tests were employed following the validation of data normality via the Shapiro-Wilk test and visual examination of Q-Q plots. Effects sizes were indicated using the partial eta square (η_2_p), with small (0.02), medium (0.13), and large (0.26) effect sizes noted [55, 56]. A threshold of *p* < .05 was used for statistical significance. If necessary, pairwise comparisons were conducted using t-tests, with the Benjamini-Hochberg procedure applied for p-value correction in multiple tests [57]. Significant effects were interpreted only when of sufficient intensity (η_2_*p* > .02). All values are presented as mean (SD) unless stated otherwise. In the absence of three-level interaction effects, only two-level interaction effects were reported for each factor combination. Note that the degrees of freedom of the analysis are varied across variables due to differing exclusion rates for subjects.

#### Tasks performance and kinematics

The movement parameters for the circular steering task (IPe, speed, accuracy, range of trunk anterior flexion, range of elbow extension) were evaluated through a mixed ANOVA, which included group (healthy and stroke) as a between-subject factor, and hand (non-paretic/dominant and paretic/non-dominant hand) as a within-subject factor. Similarly, a mixed ANOVA was employed for the paced-reaching task (PANU, mean velocity, range of trunk anterior flexion, range of elbow extension), incorporating group (healthy and stroke) as a between-subject factor and hand (non-paretic/dominant and paretic/non-dominant hand) and condition (spontaneous- SAU and maximal- MAU) as within-subject factors.

#### Cortical activations

For the analysis of fNIRS peak of ΔHbO_2_ and fEEG Alpha and Beta ESRPs, a mixed ANOVA was applied with group (healthy and stroke) as a between-subject factor, and hand (non-paretic / dominant and paretic / non-dominant hand), condition (spontaneous- SAU and maximal- MAU, paced-reaching task), and hemisphere (contralateral / ipsilesional; ipsilateral / contralesional) as within-subject factors.

#### Brain-movement relationship

In our investigation of the association between performance in the circular steering task and brain activation (fNIRS peak ΔHbO_2_) across the groups, we consistently applied Spearman rank partial correlation analysis. This approach was chosen to account for the non-normal distribution of some variables and to maintain consistency across the analysis, thus enhancing comparability of our findings. We choose to keep only moderate effects to avoid false effects, thus, we just present correlation with at least a r_s__2_ > 0.25. Only those effects were reported to facilitate the results presentation. As statistics were undertaken with a non-parametric Spearman rank correlation, no regression lines were built on the figure representing the correlations as they would be misleading.

## Results

### Tasks performance and kinematics

#### Circular steering task

**Fig. 1:**
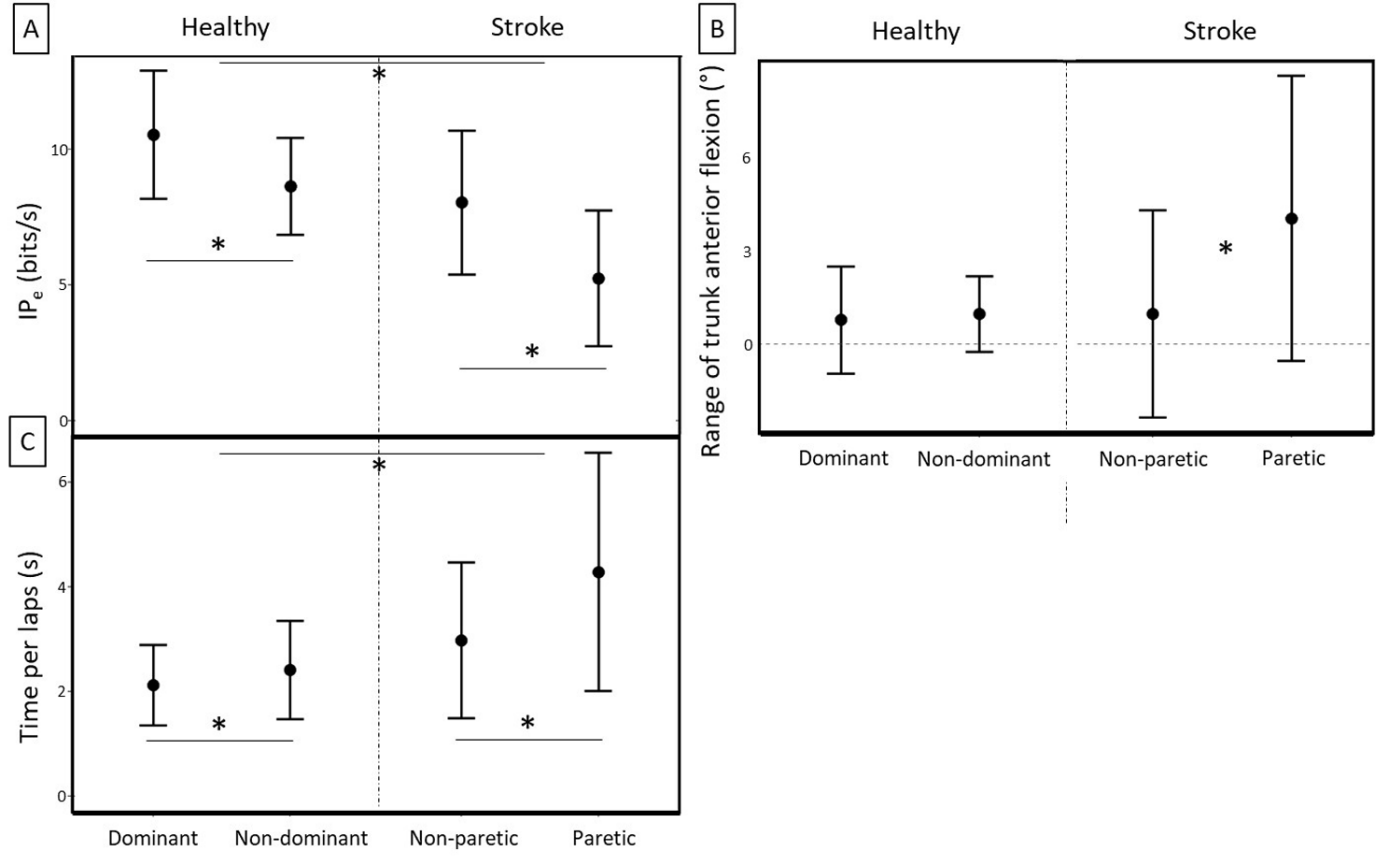
Circular steering task performances and strategies *(mean ± SD)* for the two groups and according to hand trial. **(A)** Index of performance (IP_e_); **(B)** Range of trunk anterior flexion; and **(C)** Time per lap. (* for statistically significant differences at *p* < .05)

On the circular steering task (Fig. 1), we found a higher performance (IP_e_) in the healthy group and with the dominant hand / non paretic hand for both groups (Group: F_(1,40)_ = 20.52, *p* = .000, η_2__p_ = .34; Hand: F_(1,40)_ = 53.00, *p* = .000, η_2__p_ = .57) with no Group × Hand interaction (F_(1,40)_ = 1.97, *p* = .169, η_2__p_ = .05). For the speed component (i.e., time per lap), we found a Group x Hand interaction (F_(1,40)_ = 5.83, *p* = .020, η_2__p_ = .13). Post-hoc analysis showed that the time per lap difference between paretic/non-dominant and non-paretic/dominant hand, was significantly higher for the stroke group, with a longer time per lap with the paretic arm (Healthy: η_2__p_ = .25; Stroke: η_2__p_ = .34). Moreover, it shows that the time per lap was significantly shorter in the healthy group, whatever the hand. For the accuracy component (i.e., bias), we did not find any significant effects (healthy / dominant: bias 183 (± 56.6); healthy / non-dominant: bias = 189 (± 49.5); stroke / non-paretic: bias = 202 (± 77.9); stroke / paretic: bias = 233 (± 96.3).

On the circular steering task, we found that the trunk compensations were higher in the stroke group when performing with the paretic hand (Group x Hand interaction: F_(1,35)_ = 8.95, *p* = .005, η_2__p_ = .20). For both groups, the range of elbow extension was significantly higher with the dominant / non -paretic hand (F_(1,35)_ = 8.28, *p* = .007, η_2__p_ = .19).

#### Paced reaching task

**Fig. 2:**
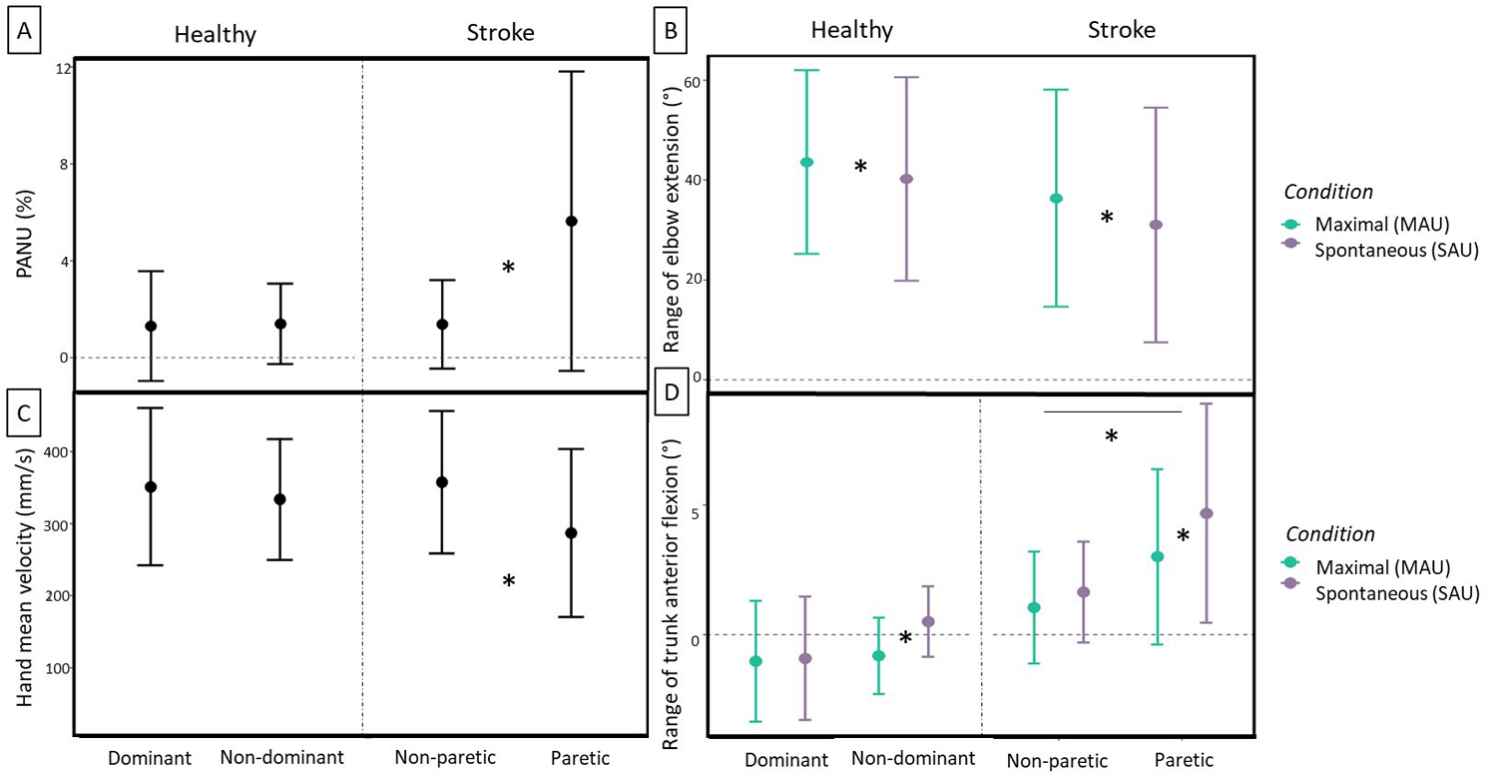
Paced reaching task upper limb movement strategies *(mean ± SD)* for the healthy and stroke groups and according to hand trial and condition (for condition interaction and/or effects). **(A)** Proximal-arm non-use, PANU; **(B)** Range of elbow extension; **(C)** Hand mean velocity; and **(D)** Range of trunk anterior flexion. (* for statistically significant differences at *p* < .05)

On the paced reaching task (see Fig. 2), we found a Group x Hand interaction on the PANU, range of trunk flexion and hand mean velocity (PANU: F _(1,37)_ = 8.85, *p* = .005, η_2__p_ = .19; range of trunk flexion: F _(1,37)_ = 5.01, *p* = .031, η_2__p_ = .12; hand mean velocity: F _(1,37)_ = 4.93, *p* = .033, η_2__p_ = .12). The range of trunk anterior flexion, and PANU were higher for the stroke paretic hand and at the same time the hand mean velocity was lower. For the range of trunk anterior flexion, we found a Hand x Condition interaction showing that the range of anterior trunk flexion was lower in the maximal condition for the non-dominant / paretic hand (F _(1,37)_ = 4.88, *p* = .033, η_2__p_ = .12). We also found a condition effect on the range of elbow extension, for both groups, it was higher in the maximal condition (F _(1,37)_ = 7.11, *p* = .011, η_2__p_ = .16).

### Brain activity

**Fig. 3:**
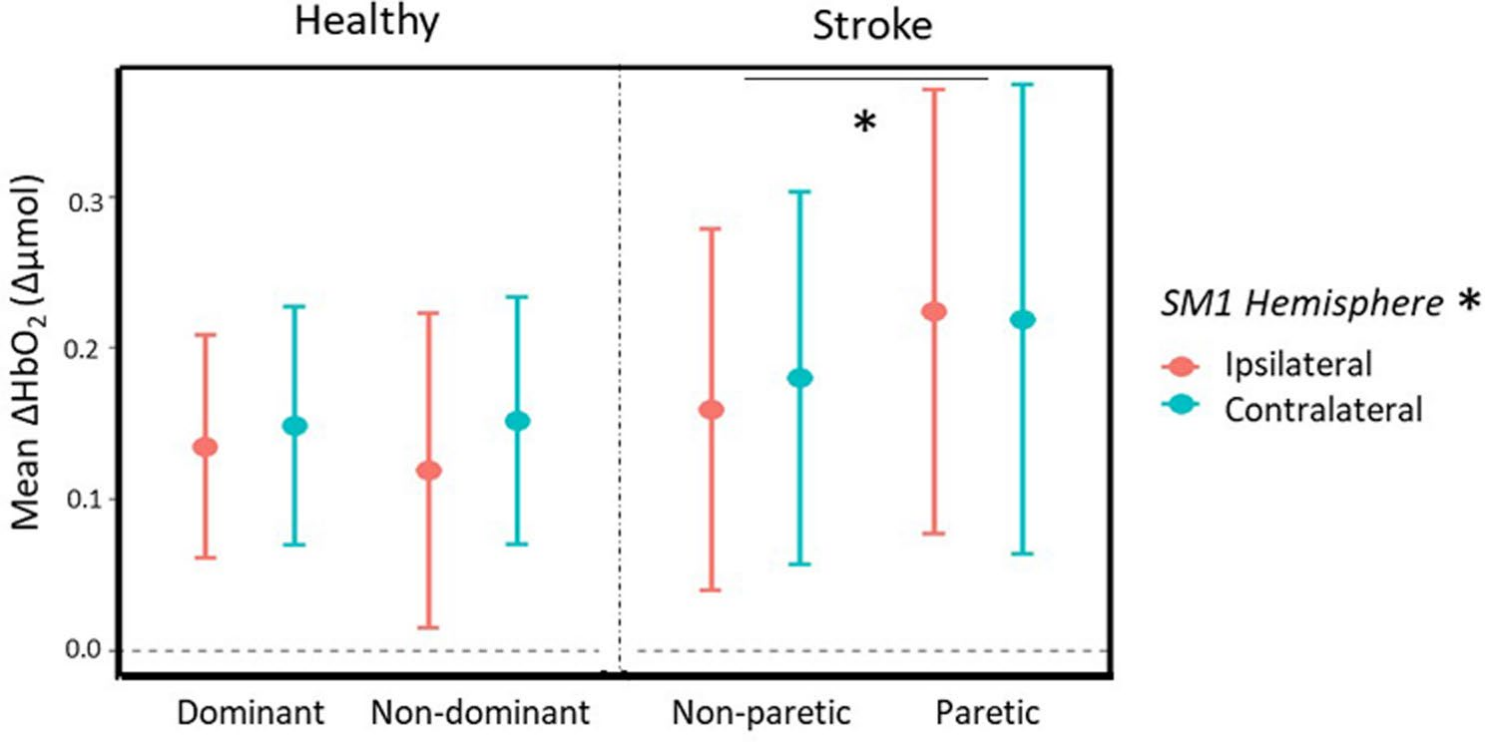
Paced reaching task fNIRS mean ΔHbO_2_ peak (mean ± SD) for the healthy and stroke groups as a function of hand and hemisphere (ipsilateral in orange; contralateral in cyan). * For statistically significant differences at *p* < .05: hand effect in the stroke group and hemisphere effect for all groups and conditions

**Fig. 4:**
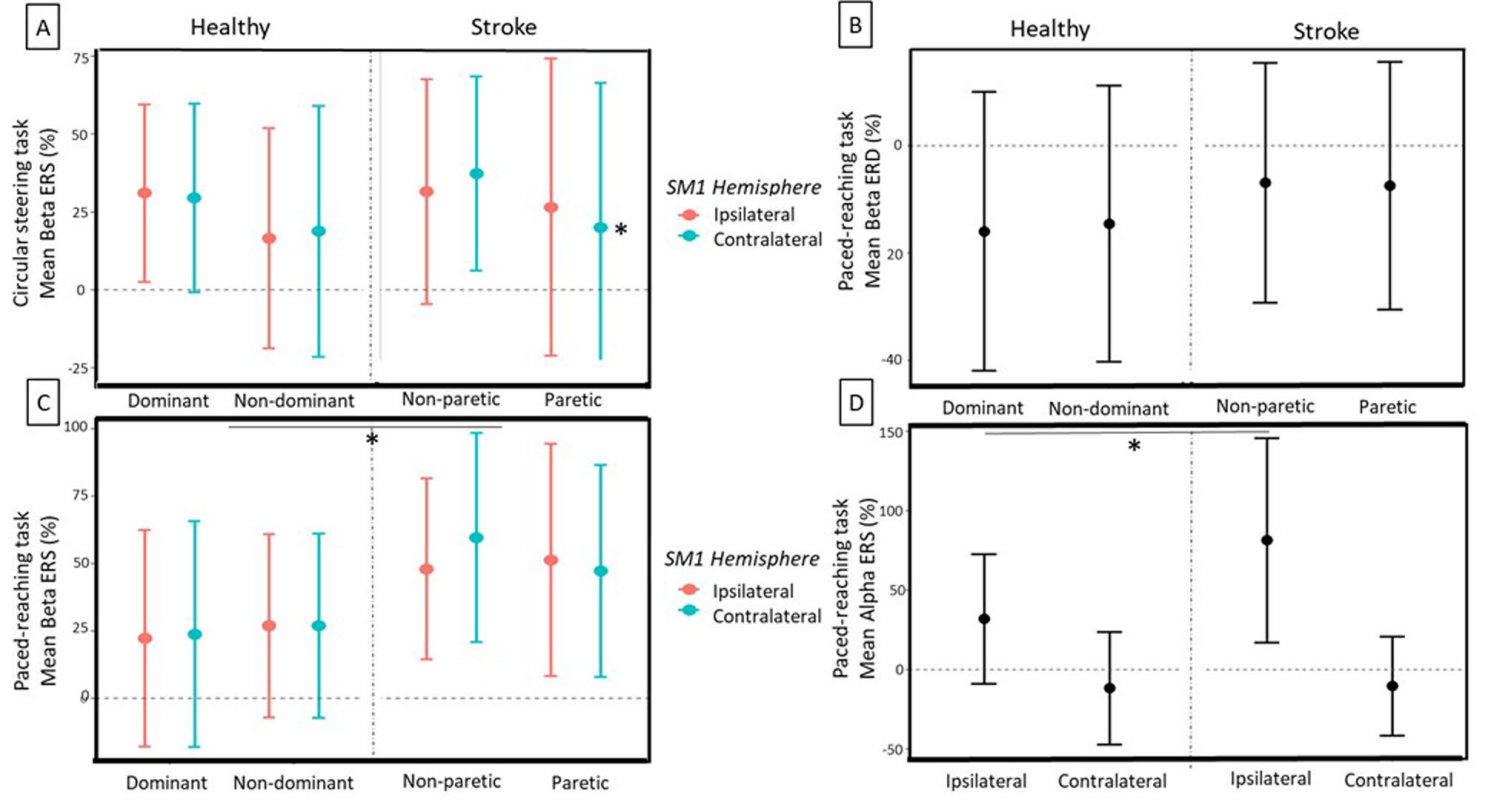
fEEG Beta event-related spectral perturbation (ERSP) *(mean ± SD)* for the healthy and stroke groups. Circular steering task: lower Beta event-related synchronization (ERS) in contralateral (ipsilesional) hemisphere of the stroke group after paretic arm movement **(A)**. Paced reaching task: Beta event-related desynchronisation (ERD) with a tendency to lower ERD in the stroke group **(B)**, higher Beta ERS in the stroke group for both hemispheres **(C)**, and a higher Alpha ERS for the stroke group in the Ipsilateral hemisphere. (* for statistically significant differences at *p* < .05)

Brain activity (fNIRS: peak of ΔHbO_2_; fEEG: ERD and ERS) during paced reaching and circular steering tasks are presented in Fig. 3 (fNIRS) and Fig. 4 (fEEG) and the statistical results are detailed in the supplementary materials for group, hand, hemisphere, and condition effects and two-way interaction effects with each factor combinations (see Supplementary material files 2 and 3). The significant three-levels interactions are reported in the text.

#### fNIRS

##### Circular steering

The analysis of the mean ΔHbO_2_ peak during the circular steering task showed no significant effects.

##### Paced reaching task

The analysis of the mean ΔHbO_2_ peak during the paced reaching task showed a higher activation for stroke group with the paretic hand (Group x Hand: F _(1,36)_ = 4.51, *p* = .041, η_2__p_ = .11) and a higher activation in the contralateral side compared to the ipsilateral one for both groups (Hemisphere: F _(1,36)_ = 6.45, *p* = .016, η_2__p_ = .15). Nevertheless, the three-way interaction Group x Hand x Hemisphere (F _(1,36)_ = 2.82, *p* = .102, η_2p_ = .07) showed a trend for difference between the two hemispheres or the ipsilateral (contralesional) hemisphere being higher than the contralateral (ipsilesional) side for paretic hand use in the stroke group.

#### fEEG

##### Circular steering task

On the circular steering task (Fig. 4A), we found for Beta ERS a 3-way interaction Group x Hand x Hemisphere (F _(1,25)_ = 5.02, *p* = .034, η_2__p_ = .17). Post-hoc comparisons revealed that, for the stroke group, there was a Hand x Hemisphere interaction (F _(1,14)_ = 7.56, *p* = .016, η_2__p_ = .35) showing a lower post-movement synchronization in the contralateral (ipsilesional) hemisphere when performing with the paretic hand (see Fig. 4A). The analysis of the mean ERSP did not show any main or interaction effect of Group on Alpha and Beta ERD nor Alpha ERS.

##### Paced reaching task

On the paced reaching task, there was a Group x Brain interaction for the Beta ERD (F _(1,23)_ = 4.98, *p* = .036, η_2__p_ = .19). Although there was a tendency to a Group effect (F _(1,29)_ = 3.88, *p* = .051, η_2__p_ = .03) showing a smaller Beta desynchronization in the stroke group (Fig. 4B), the post-hoc comparisons between the different modalities of the Group x Brain interaction were too low to emerge, and thus are not shown in Fig. 4B. For the Alpha ERD we did not find any significant main or interaction effect. For the Beta ERS, we found a Hand x Condition interaction showing that for the stroke group, the post-movement Beta synchronization was higher for the maximal condition (F _(1,21)_ = 8.80, *p* = .007, η_2__p_ = .30; see Fig. 4C). We also found a Group x Hand x Hemisphere interaction (F _(1,21)_ = 5.08, *p* = .035, η_2__p_ = .20). Post-hoc comparison revealed a Hand x Brain interaction for the stroke group (F _(1,27)_ = 14.9, *p* = .001, η_2__p_ = .36) showing a higher Beta ERS with the dominant / non-paretic hand in the stroke group. For Alpha ERS, we found a Group x Hemisphere interaction (F _(1,16)_ = 4.53, *p* = .049, η_2__p_ = .22). Post-hoc comparisons revealed that there was a Group effect in the ipsilateral hemisphere (F _(1,53)_ = 28.8, *p* = .000, η_2__p_ = .35), showing a higher post-movement Alpha synchronization in the ipsilateral hemisphere of the stroke group in comparison to the healthy group (see Fig. 4D). There was also a Hand x Hemisphere interaction (F _(1,16)_ = 6.28, *p* = .023, η_2__p_ = .28). Post-hoc comparisons revealed a Hand effect in the contralateral hemisphere (F _(1,51)_ = 4.70, *p* = .035, η_2__p_ = .08), with a higher post-movement synchronization in the non-dominant / paretic hand.

### Brain-movement-clinical scores relationship in the stroke group

#### Brain-movement relationship

##### Circular steering task

**Fig. 5:**
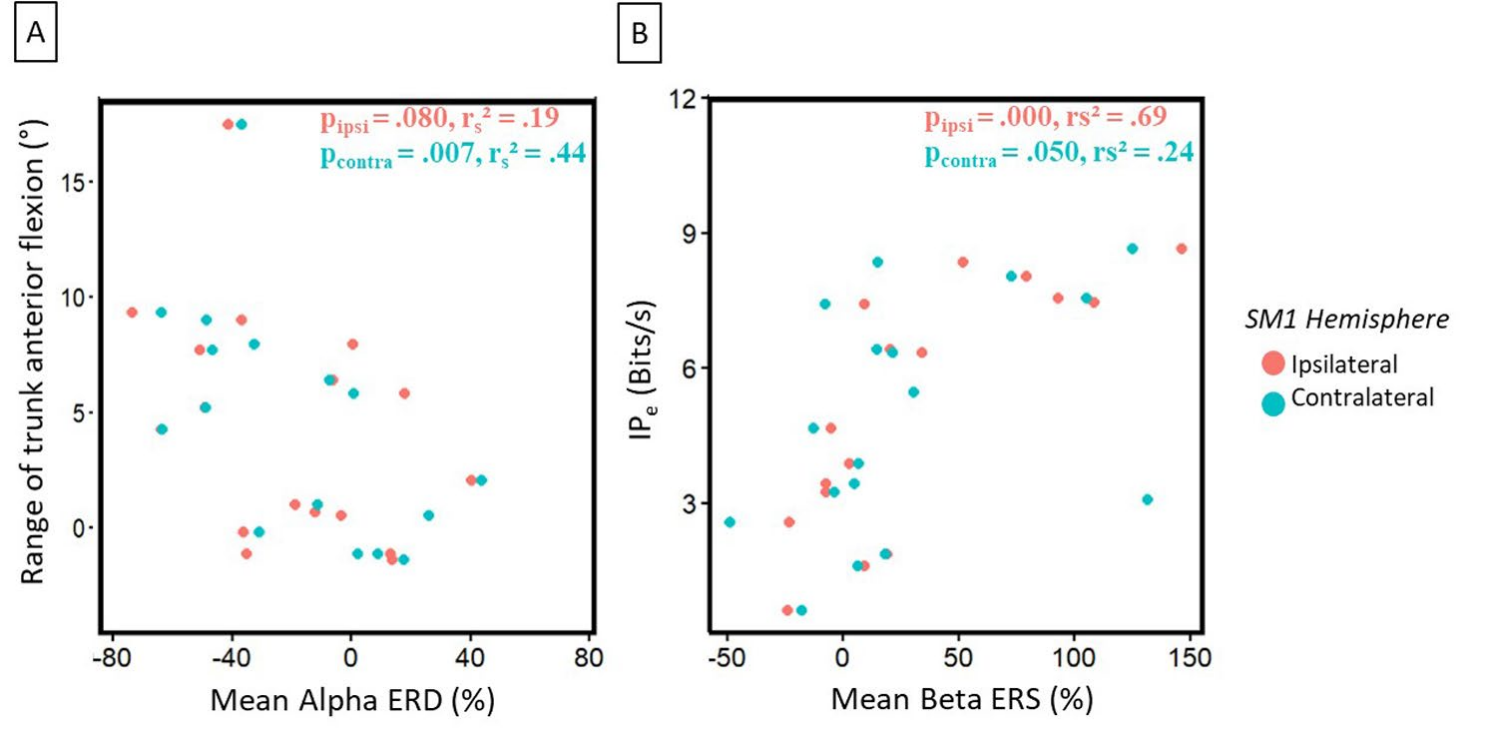
Stroke group correlation between **(A)** Alpha ERD and the trunk use, and **(B)** Beta ERS and the index of effective performance (IP_e_) during the circular steering task with the paretic arm

The spearman rank correlation analysis for the circular steering task with paretic hand showed that an increased use of the trunk was associated with a higher movement Beta desynchronization on the contralateral (ipsilesional) hemisphere (*p* = .007, r_s__2_ = 0.44) and a tendency in the ipsilateral (contralesional) side (*p* = .080, r_s__2_ = 0.19, see Fig. 5A). We also found that for a higher IP_e_ and time per lap in the circular steering task, there was a higher post-movement Beta synchronization in the ipsilateral (contralesional) hemisphere and a tendency in the contralateral one (IP_e_ - Ipsilateral: *p* = .000, r_s__2_ = 0.69; Contralateral: *p* = .050, r_s__2_ = 0.24; Time per lap – Ipsilateral: *p* = .000, r_s__2_ = 0.49; Contralateral: *p* = .031, r_s__2_ = 0.28, see Fig. 5B).

##### Paced reaching task

**Fig. 6:**
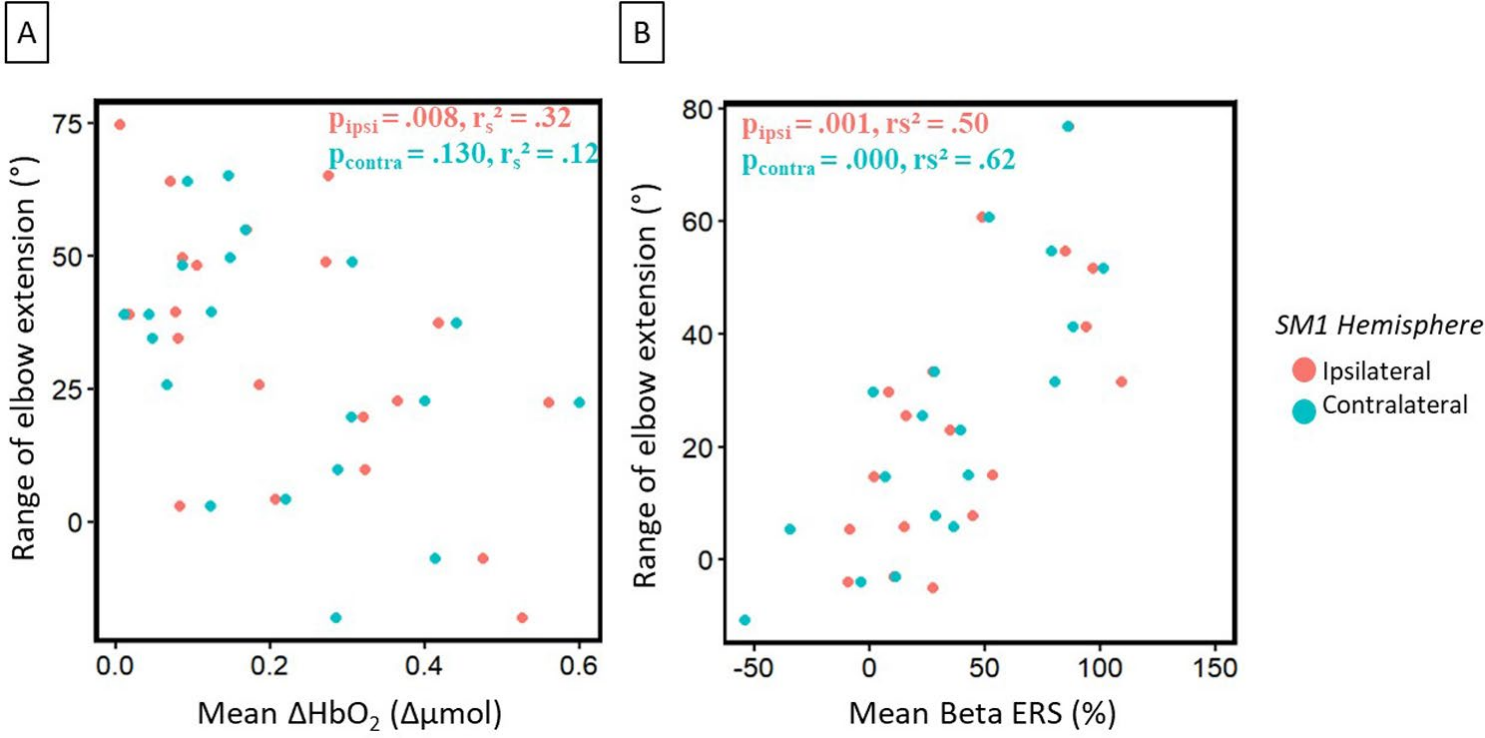
Stroke group correlation between **(A)** Peak of ΔHbO_2_ and elbow extension and **(B)** Mean Beta ERS and elbow extension during the paced-reaching task with the paretic arm

When reaching in the maximal condition, we found that elbow extension was negatively correlated with the ipsilateral hemisphere (contralesional) peak of ΔHbO_2_ (*p* = .008; r_s__2_ = 0.32, see Fig. 6A) but not for with the contralateral one (*p* = .130; r_s__2_ = 0.12). We also found that the slower to do the maximal reaching have a higher post-movement synchronization in the ipsilateral hemisphere (*p* = .003, r_s__2_ = 0.47). We found that for a higher spontaneous elbow extension the Beta post-movement synchronisation was higher in both hemispheres (Ipsilateral: *p* = .001, r_s__2_ = 0.50; Contralateral: *p* = .000, r_s__2_ = 0.62, see Fig. 6B). On the same conditions, the Alpha post-movement synchronization in the ipsilateral hemisphere was also positively correlated to elbow extension (*p* = .009, r_s__2_ = 0.37).

#### Brain-clinical scores relationship

**Fig. 7:**
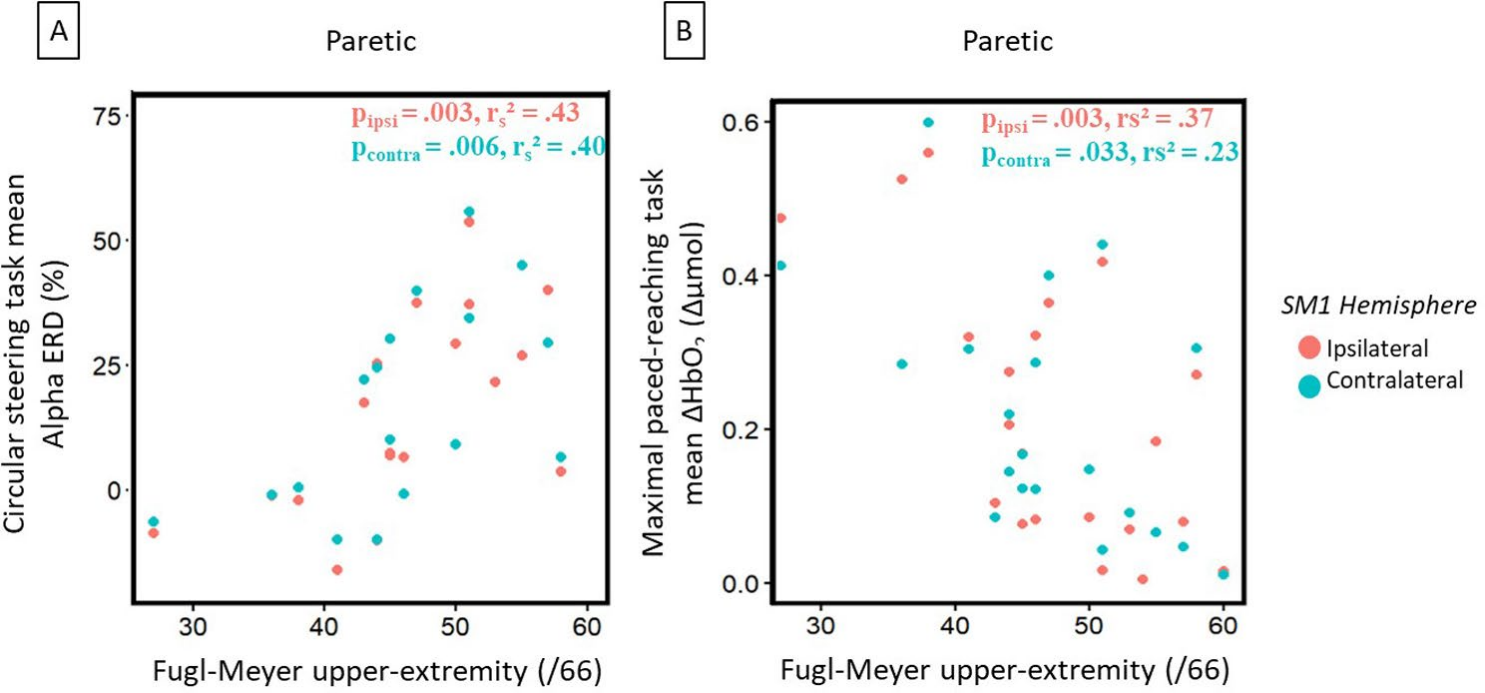
Stroke group correlation between **(A)** Alpha ERD during the circular steering task and the FM-UE test and, **(B)** Peak of ΔHbO_2_ during the maximal paced-reaching task and the FM-UE test

For the correlation between the brain parameters and the clinical scores, we found that a more marked Alpha ERD on the circular steering task was associated to a lower FM-UE (Non-paretic hand - Ipsilateral: *p* = .000, r_s__2_ = 0.62; Non-paretic hand – Contralateral: *p* = .000, r_s__2_ = 0.55; Paretic hand - Ipsilateral: *p* = .003, r_s__2_ = 0.43; Paretic hand – Contralateral: *p* = .006, r_s__2_ = 0.40, see Fig. 7A).

For the maximal condition of the reaching task with the paretic hand, we found a negative correlation between the peak of ΔHbO_2_ and FM-UE, showing that for a better clinical score there was a lower ipsilateral (contralesional; *p* = .003, r_s__2_ = 0.37) and contralateral (ipsilesional: *p* = .033, r_s__2_ = 0.23) peak of ΔHbO_2_ (see Fig. 7B).

## Discussion

This study investigated the impact of chronic stroke on the bilateral SM1 electrical (fEEG) and hemodynamic (fNIRS) responses during unilateral proximal UL movements. We concurrently recorded bilateral SM1 activity via combined fNIRS/ fEEG, along with UL movements using kinematic tracking. Two previously established UL functional tasks were employed: a paced-reaching task and a circular steering task designed to interrogate the speed-accuracy trade-off [16, 28]. Our main finding was a greater increase in bilateral SM1 activity (fNIRS-peak ΔHbO_2_) for the paretic UL than non-paretic one during the paced-reaching task. Regarding the movement modifications, we observed that stroke patients showed slower speeds, increased trunk compensation, and decreased spontaneous use of the elbow-shoulder joint, particularly on the paretic side.

For the paced-reaching task, a greater increase in bilateral SM1 activation was detected with fNIRS during the movement with the paretic hand which tended to coincide with reduced fEEG Beta desynchronization at the onset of movement. These results might indicate compensatory brain mechanisms designed to mitigate the effects of stroke on movement execution. The fEEG findings presented a lower SM1 excitability in the stroke group, which was associated to an increased activation of the fNIRS SM1 when moving the paretic hand. This aligns with previous studies suggesting reduced brain asymmetry and increased activity as potential mechanisms of post-stroke motor recovery [58, 59]. These findings can also be compared to the study by [18] which analysed fNIRS activity during a modified Box & Block forward reaching test in stroke patients compared to a healthy cohort. Despite the inferior performance, stroke patients demonstrated increased lesioned SM1 activity during paretic arm reaching.

For the circular steering task, our findings indicated a reduced performance in the stroke group, while task-related fNIRS peak and fEEG-ERD remained comparable across both groups. This task relies heavily on visuomotor control: continuous monitoring and rectification of the trajectory while moving as fast as possible, embodying the concept of the continuous speed-accuracy trade-off [60]. Given these extensive requirements on sensorimotor control networks, this task is a good measure of neural efficiency, i.e., the amount of neural resources required to execute a given task [61]. In this context, the hypothesis of neural efficiency postulates that individuals with higher cognitive ability exhibit lower energy consumption in the brain for equivalent tasks [62]. Given the decreased performance in the stroke cohort and similar brain activity levels, it could be inferred that these individuals exhibit reduced neural efficiency when performing the circular steering task. However, as our study was confined to the SM1 region, we cannot draw a definitive conclusion regarding overall neural efficiency. Indeed, the circular steering task demands a significant level of visuomotor control, and previous research has suggested that the prefrontal area plays a substantial role in controlling such movements [63]. However, despite the potential impairment of neural efficiency in SM1, the absence of significant brain modification in stroke patients may be explained by considering the circular steering task’s nature. Indeed, the task required maximal performance from the healthy subjects as well. Moreover, our previous study found no effects of healthy aging on the level of fNIRS SM1 activity in this task, as older adults engaged both SM1 to compensate for their reduced neural efficiency [16]. We can thus hypothesize that when performance is maximized (“as fast as possible”) for all participants, brain activity will reach its maximum, and compensatory mechanisms may rely on alternative neural pathways, such as the prefrontal areas [63]. Our previous work also indicated no significant effects of healthy aging on any brain or kinematics parameters during the reaching task. This lack of effect underscores the notion that, the reaching task was, for our healthy adults, considerably simpler and less demanding than the circular steering task. However, in the present study, we observed modifications in brain activity during the paced-reaching task, potentially due to its complexity for post-stroke patients demonstrating motor compensation to complete the task successfully as in the circular steering task.

Focusing on the stroke-induced alterations in movement control, we observed the deployment of compensatory strategies by stroke patients to accomplish both functional tasks using their paretic UL. Specifically, in the circular steering task, stroke patients employed their trunk to facilitate task completion with their paretic hand, concomitantly showing reduced use of the elbow-shoulder joints. Similarly, during the reaching task, we detected evidence of proximal-arm non-use (i.e., non-mandatory trunk compensation) when the task was performed with the paretic hand. Additionally, the velocity of the paretic hand was reduced, a finding of particular interest given our use of paced reaching, indicating that the stroke patients were moving slowly to follow the paced rhythm. This result could be explained by the existence of strong correlations between clinical scores and velocity implying that the patients’ movement difficulties may be attributed to their level of impairment. This observation is consistent with prior studies demonstrating that the speed of the paretic movement is slower than that of the non-paretic movement [64]. This also aligns with our findings from the circular steering task, indicating reduced movement speed in the stroke group, particularly for the paretic arm.

A secondary aim of this study was to explore the association between brain and movement kinematics. These results are a first step for a better understanding of the underlying mechanisms of post-stroke motor recovery but as exploratory have to be treated with caution. We identified an association between trunk use and fEEG Alpha desynchronization in the circular steering task. This could imply that trunk use necessitates mobilizing increased neuronal resources across both hemispheres. Further, we detected alterations in post-movement Beta synchronization associated with motor performance. Specifically, a higher IP_e_ correlated with increased ERS. We could hypothesize that, in this task, the high demand level is sustained by the highest-performing subjects, who are also likely to move fastest. It is well established that increased speed correlates with higher neural activity [65], implying that the ratio between the movement and rest period could be higher. Regarding the reaching task, we observed different effects depending on whether the task was performed spontaneously or maximally. In the spontaneous reaching task, our findings mirror those of the circular task, with higher post-movement synchronization observed in better performers. Conversely, the positive correlation between movement time and Alpha ERS in the maximal condition is more challenging to explain. One could hypothesize that the enhanced synchronization for slower performers might be explained by the extended duration of neural demand they experience during the task. As they move slower, their SM1 will be engaged for a longer time (i.e., the paced reaching task typically entails 2s of movement and 2s of rest), leading to higher synchronization in the ERSP. Nevertheless, the negative correlation between fNIRS brain activity and elbow extension could be akin to the circular steering task, could suggest an over-activation in lower performers who engage their trunk to facilitate movement. Another hypothesis could be that in the maximal condition, we instruct patients to use their elbow-shoulder joints maximally. Consequently, those who employ these joints less frequently will likely require more resources and increased brain activity. Thus, we could observe either the effect of trunk use or the effect of effort. However, our measurements cannot discern which hypothesis is closer to the truth (i.e., a measure of perceived effort could have been beneficial).

Lastly, an exploratory aim of this study was to investigate the association between stroke patients’ clinical scores and the corresponding brain parameters. We observed meaningful correlations that underscore the potential of fNIRS and fEEG methodologies in the context of stroke rehabilitation [18, 66, 67]. First, we found that a more pronounced Beta desynchronization at movement was linked to a lower score on the FM-UE. It is in line with prior research illustrating that a more significant event-related desynchronization in the sensorimotor cortex correlates with an enhanced demand for concentration and excitatory drive of pyramidal cells during task execution [68]. For example, studies on grip tasks during rehabilitation have shown that with progression and motor improvement, there is a reduced requirement for cortical engagement and effort to perform the grip task [69]. Secondly, the inverse correlation between fNIRS brain activity and FM-UE indicates that a lower clinical score corresponds with an increased SM1 activation during the execution of the paced-reaching task. This is plausible considering the kinematics of the task. Indeed, we found an association between higher elbow extension and higher WMFT scores (data not shown). Which could suggest that patients who utilize their arm extension more during the reaching task will have higher clinical scores, and conversely for patients using more trunk compensation to do the task. It is also known that elbow extension negatively correlates with trunk compensation [28, 47]. Consequently, patients with greater upper limb deficits may rely more on their trunk to reach the target, leading to larger brain activity in response to the increased demand for the trunk.

The methodology for the seated reaching and circular steering tasks proposed in this paper, including joint kinematics assessment of UL proximal movements and brain SM1 activity, seems well-suited for a pathological population. The combined fEEG and fNIRS methods provide detailed information about the neural and hemodynamic mechanisms underlying movement [70, 71]. Moreover, using these two controlled tasks allows for an ecological evaluation of movement within the context of functional recovery, enabling an assessment as close as possible to daily living activities [29, 43, 47]. And the analysis of movement parameters selected, such as speed, accuracy, and compensation strategies, could indicate the evolution of motor recovery [33]. Moreover, as previous studies suggest using brain laterality as an indicator of motor recovery [72], our evaluation method could be useful in routine assessments to better characterize patients’ conditions. In this study, we identified differences at the level of the kinematics and of the brain suggesting that the method developed was suitable for evaluating the stroke effects. Additionally, by combining these tools, we identified correlations between brain parameters, movement kinematics, and clinical scores. For example, in this paper, we found a brain/movement correlation for trunk use, which is important in post-stroke rehabilitation evaluation [33]. This approach might eventually allow us to identify neural markers of trunk compensation or other movement strategies, though more studies are needed to confirm this. The objective of the present study was to evaluate the effects of stroke on the brain and kinematic strategies using a newly developed method. As such, we currently lack sufficient information to provide prognostic strategies for motor rehabilitation, indeed knowing the change in the brain and kinematics following rehabilitation is necessary to answer this problem. Nevertheless, we hypothesize that this method will enable a deeper evaluation of the effects of rehabilitation methods used in clinical settings. For example, the currently running ReArm project is using this method to evaluate the effects of transcranial electrical brain stimulation (HD-tDCS) and virtual reality therapy on post-stroke upper-limb motor recovery [40].

This study has several limitations. Firstly, while age and gender matching were not strictly adhered to, the differences observed should not significantly impact the results. Specifically, the gender ratio was more balanced in the healthy group compared to the stroke group (healthy group: 11 women, stroke group: 8 women). However, statistical analysis showed no significant gender difference between the groups (chi-square test, *p* = .116). Therefore, gender is not considered a limitation affecting our findings. Previous literature [73–75] and our prior work [16] further support that sex does not significantly influence the variables we study (fEEG ERD-ERS; fNIRS) in the context of the functional tasks examined. Regarding age, there is a significant difference between the groups (mean age for healthy group = 72 years, stroke group = 64 years; t-test, *p* = .002). However, the stroke group is younger on average than the healthy group. This is relevant because the effects we are investigating, such as the decline in performance and lateralization, are typically associated with aging. Thus, the younger average age in the stroke group would likely reduce, rather than exaggerate, the differences between the groups. Moreover, correlation analyses showed no significant relationship between age and the studied variables, indicating that the age range is insufficient to show age effects on these variables, supporting our decision not to include age as a covariate in the ANOVA models. Finally, the available literature is indicating that age is not a predictor of the functional recovery [76–78], particularly our previous work did not show any effect of age on most of the brain variables studied here [16]. Additionally, in this study we did not take into account the role of associated cognitive disorders (in particular visuospatial disorders, for example, which certainly interfere a great deal with the circular task) and sensory disorders (also very important for the circular task, which relies heavily on proprioception), as well as spasticity, which interferes a great deal with elbow extension and compensatory movements by the trunk. Lastly, the reaching task, paced at a consistent rhythm for all participants, could present a significant limitation. This speed constraint could lead to an augmented use of compensatory movements in stroke patients to reach the ball at the required speed [79].

## Conclusion

In conclusion, this study provides insight into the impacts of stroke on task-related brain activity and kinematics during unilateral upper limb movements that engage full UL joint movements (i.e., shoulder, elbow, wrist). Our findings highlight the brain and movement compensations associated with a chronic post-stroke population. Additionally, we demonstrate the utility of a combined fNIRS-fEEG recording approach, which correlates with kinematic and clinical scores. The concurrent evaluation of brain and kinematic parameters in ecological settings offers complementary information about the execution of paretic movements, allowing for extracting specific components for targeted intervention during rehabilitation. Moreover, these measures can enrich routine clinical assessments in ecological settings. As perspectives, the ReArm project, of which this study is a part, aims to discern the effects of rehabilitation on these specific brain and kinematic parameters. Furthermore, we aim to investigate their applicability in routine evaluation to facilitate more personalized rehabilitation strategies.

## Electronic supplementary material

Below is the link to the electronic supplementary material.

Supplementary Material 1: Flowchart of the fEEG and fNIRS pre-processing and processing steps. Method based on previous study. (Muller 2023). *Abbreviations* ERSP, event-related spectral perturbation; ERS, event-related synchronization; ERD, event-related desynchronization; RP, relative power; P_n_, power spectrum; ΔHbO_2_, variation of oxygenated blood

Supplementary Material 2: Statistical results of the ANOVA on the fEEG and fNIRS brain parameters for the circular steering task

Supplementary Material 3: Statistical results of the ANOVA on the fEEG and fNIRS brain parameters for the paced-reaching task

## Data Availability

Data will be available from the corresponding author on reasonable request.

## Abbreviations

BBT: Box and Block Test
BI: Barthel Index
ERD: Event Related Desynchronization
ERS: Event Related Synchronization
ERSP: Event Related Spectral Perturbation
FM-UE: Fugl-Meyer Upper-Extremity
IDe: Index of Task Effective Difficulty
IPe: Index of effective performance
fEEG: Functional Electroencephalography
fNIRS: Functional Near-infrared Spectroscopy
HbO2: Oxygenated blood
HbR: Deoxygenated blood
MAU: Maximal Arm Use
PANU: Proximal Arm Non-Use
SAU: Spontaneous Arm Use
SD: Standard Deviation
SM1: Primary Sensorimotor cortex
WMFT: Wolf Motor Function Test
