# Supplementary figures and images for: Brain-movement relationship during upper-limb functional movements in chronic post-stroke patients
Source: J Neuroeng Rehabil. 2024 Oct 22;21:188. doi: 10.1186/s12984-024-01461-3 (PMC11494975; doi:10.1186/s12984-024-01461-3)

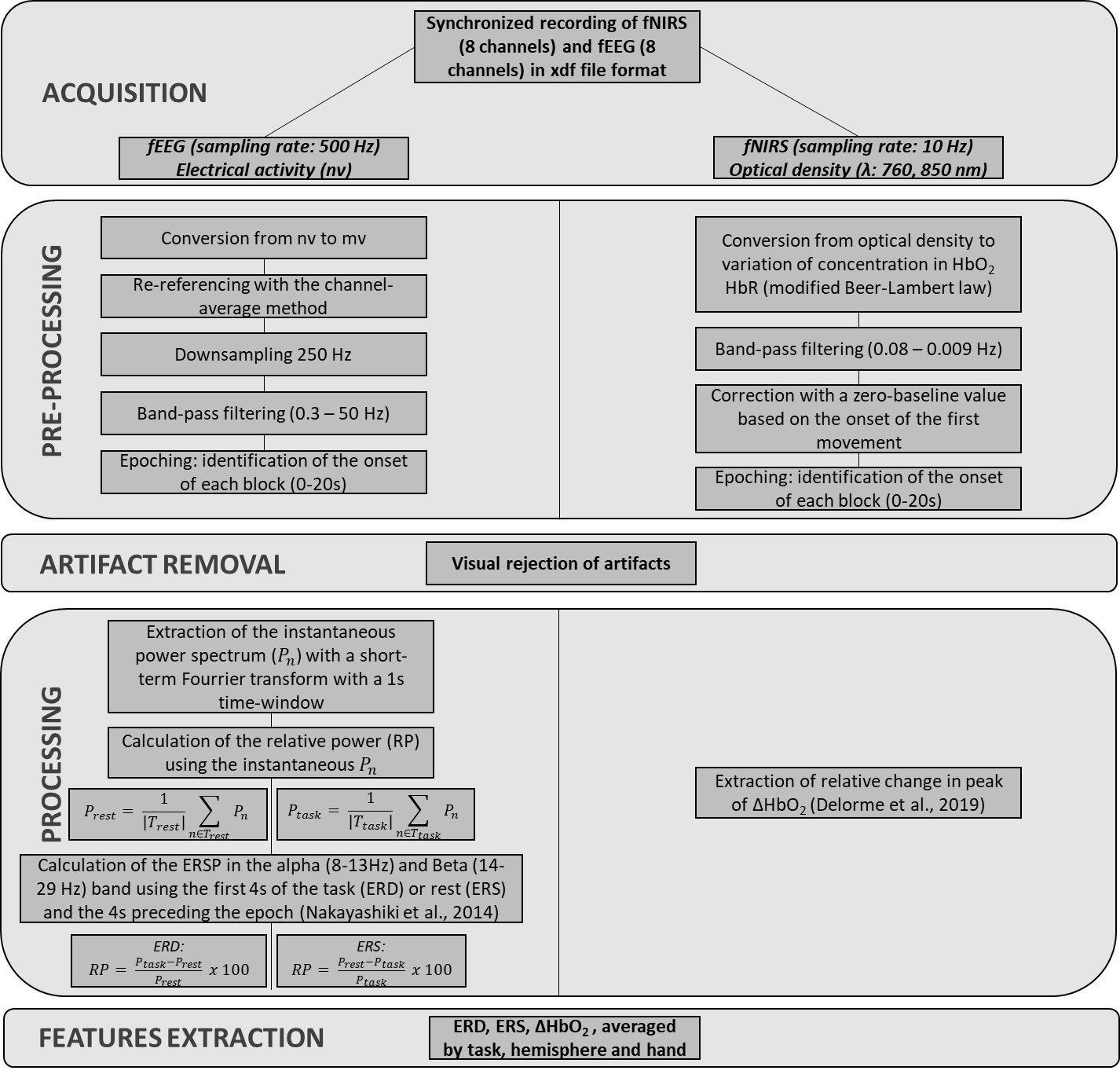

Supplement: Supplementary file 1 — Supplementary Material 1: Flowchart of the fEEG and fNIRS pre-processing and processing steps. Method based on previous study. (Muller 2023). Abbreviations ERSP, event-related spectral perturbation; ERS, event-related synchronization; ERD, event-related desynchronization; RP, relative power; Pn, power spectrum; ΔHbO2, variation of oxygenated blood [file 12984_2024_1461_MOESM1_ESM.jpg]
