# Supplementary material for: Brain-movement relationship during upper-limb functional movements in chronic post-stroke patients
Source: J Neuroeng Rehabil. 2024 Oct 22;21:188. doi: 10.1186/s12984-024-01461-3 (PMC11494975; doi:10.1186/s12984-024-01461-3)
Supplement: Supplementary file 2 — Supplementary Material 2: Statistical results of the ANOVA on the fEEG and fNIRS brain parameters for the circular steering task [file 12984_2024_1461_MOESM2_ESM.pdf]

**Additional file 2.** Statistical results of the ANOVA on the fEEG and fNIRS brain parameters for the circular steering task.

| **A.** | **GROUP** | **HEMISPHERE** | **HAND** |
|---|---|---|---|
| fEEG | | | |
| Alpha ERD | F (1, 27) = 0.66, p = .424, $\eta^2_p$ = .02 | F (1, 27) = 1.45, p = .293, $\eta^2_p$ = .04 | F (1, 27) = 0.42, p = .523, $\eta^2_p$ = .01 |
| Beta ERD | F (1, 27) = 0.08, p = .780, $\eta^2_p$ = .00 | F (1, 27) = 0.21, p = .654, $\eta^2_p$ = .01 | F (1, 27) = 0.40, p = .534, $\eta^2_p$ = .01 |
| Alpha ERS | F (1, 25) = 1.28, p = .269, $\eta^2_p$ = .05 | F (1, 25) = 0.00, p = .956, $\eta^2_p$ = .00 | F (1, 25) = 2.76, p = .109, $\eta^2_p$ = .09 |
| Beta ERS | F (1, 25) = 0.15, p = .698, $\eta^2_p$ = .01 | F (1, 25) = 0.00, p = .996, $\eta^2_p$ = .00 | F (1, 25) = 2.45, p = .127, $\eta^2_p$ = .09 |
| *fNIRS* | | | |
| $\Delta HbO_2$ peak | F (1, 31) = 0.00, p = .967, $\eta^2_p$ = .00 | F (1, 31) = 0.20, p = .659, $\eta^2_p$ = .00 | F (1, 31) = 0.45, p = .506, $\eta^2_p$ = .01 |
| **B.** | **GROUP x HEMISPHERE** | **GROUP x HAND** | **HAND x HEMISPHERE** |
| fEEG | | | |
| Alpha ERD | F (1, 27) = 0.00, p = .999, $\eta^2_p$ = .00 | F (1, 27) = 1.04, p = .317, $\eta^2_p$ = .04 | F (1, 27) = 1.78, p = .193, $\eta^2_p$ = .06 |
| Beta ERD | F (1, 27) = 0.56, p = .461, $\eta^2_p$ = .02 | F (1, 27) = 0.90, p = .351, $\eta^2_p$ = .03 | F (1, 27) = 0.25, p = .619, $\eta^2_p$ = .01 |
| Alpha ERS | F (1, 25) = 0.26, p = .614, $\eta^2_p$ = .01 | F (1, 25) = 0.00, p = .961, $\eta^2_p$ = .00 | F (1, 25) = 0.07, p = .792, $\eta^2_p$ = .00 |
| Beta ERS | F (1, 25) = 0.03, p = .868, $\eta^2_p$ = .00 | F (1, 25) = 0.00, p = .924, $\eta^2_p$ = .00 | F (1, 25) = 1.42, p = .244, $\eta^2_p$ = .05 |
| *fNIRS* | | | |
| $\Delta HbO_2$ peak | F (1, 31) = 0.07, p = .791, $\eta^2_p$ = .02 | F (1, 31) = 1.40, p = .246, $\eta^2_p$ = .04 | F (1, 31) = 3.52, p = .070, $\eta^2_p$ = .10 |

**A.** Effects of Group (Old healthy vs Stroke), Hemisphere (Contralateral vs Ipsilateral) and Hand (Dominant / Non-paretic vs Non-dominant / Paretic) on the fNIRS and fEEG parameters on the circular steering task and **B.** Two levels interactions of the model. In bold, results with p < .05 and $\eta^2_p$ > .0
