# Supplementary material for: Brain-movement relationship during upper-limb functional movements in chronic post-stroke patients
Source: J Neuroeng Rehabil. 2024 Oct 22;21:188. doi: 10.1186/s12984-024-01461-3 (PMC11494975; doi:10.1186/s12984-024-01461-3)
Supplement: Supplementary file 3 — Supplementary Material 3: Statistical results of the ANOVA on the fEEG and fNIRS brain parameters for the paced-reaching task [file 12984_2024_1461_MOESM3_ESM.pdf]

Additional file 3. Statistical results of the ANOVA on the fEEG and fNIRS brain parameters for the paced-reaching task.

**A.**	**GROUP**	**HEMISPHERE**	**HAND**	**CONDITION**
fEEG				
Alpha ERD	F (1, 23) = 0.00, p = .997, η^2_p = .00	F (1, 23) = 0.08, p = .770, η^2_p = .00	F (1, 23) = 0.29, p = .145, η^2_p = .09	F (1, 23) = 0.49, p = .492, η^2_p = .02
Beta ERD	F (1, 23) = 2.81, p = .107, η^2_p = .11	F (1, 23) = 0.02, p = .896, η^2_p = .00	F (1, 23) = 0.01, p = .922, η^2_p = .00	F (1, 23) = 0.12, p = .734, η^2_p = .01
Alpha ERS	**F (1, 16) = 8.62, p = .010, η^2_p = .35**	**F (1, 16) = 52.21, p = .000, η^2_p = .77**	F (1, 16) = 0.06, p = .809, η^2_p = .00	F (1, 16) = 0.01, p = .927, η^2_p = .00
Beta ERS	**F (1, 21) = 5.44, p = .030, η^2_p = .21**	F (1, 21) = 2.46, p = .132, η^2_p = .11	F (1, 21) = 00.25, p = .621, η^2_p = .01	F (1, 21) = 1.24, p = .278, η^2_p = .06
fNIRS				
ΔHbO_2 peak	**F (1, 36) = 5.93, p = .020, η^2_p = .14**	**F (1, 36) = 1.45, p = .016, η^2_p = .15**	F (1, 36) = 2.98, p = .093, η^2_p = .07	F (1, 36) = 1.49, p = .231, η^2_p = .04
**B.**	**GROUP x HEMISPHERE**	**GROUP x HAND**	**GROUP x CONDITION**	**HAND x CONDITION**
fEEG				
Alpha ERD	F (1, 23) = 0.50, p = .828, η^2_p = .02	F (1, 23) = 0.08, p = .776, η^2_p = .00	F (1, 23) = 0.05, p = .828, η^2_p = .06	F (1, 23) = 0.23, p = .634, η^2_p = .01
Beta ERD	**F (1, 23) = 4.98, p = .036, η^2_p = .18**	F (1, 23) = 0.05, p = .831, η^2_p = .00	F (1, 23) = 0.82, p = .376, η^2_p = .00	F (1, 23) = 0.08, p = .786, η^2_p = .00
Alpha ERS	**F (1, 16) = 4.53, p = .049, η^2_p = .22**	F (1, 16) = 0.39, p = .539, η^2_p = .02	F (1, 16) = 0.39, p = .541, η^2_p = .02	F (1, 16) = 0.15, p = .704, η^2_p = .00
Beta ERS	F (1, 21) = 1.83, p = .191, η^2_p = .08	F (1, 21) = 1.36, p = .257, η^2_p = .06	F (1, 21) = 2.80, p = .109, η^2_p = .12	**F (1, 21) = 8.80, p = .007, η^2_p = .29**
fNIRS				
ΔHbO_2 peak	F (1, 36) = 1.98, p = .168, η^2_p = .05	**F (1, 36) = 4.51, p = .041, η^2_p = .11**	F (1, 36) = 1.52, p = .699, η^2_p = .00	F (1, 36) = 2.72, p = .108, η^2_p = .07
	**HAND x HEMISPHERE**	**HEMISPHERE x CONDITION**		
fEEG				
Alpha ERD	F (1, 23) = 1.57, p = .223, η^2_p = .06	F (1, 23) = 0.59, p = .451, η^2_p = .02		
Beta ERD	F (1, 23) = 1.04, p = .320, η^2_p = .00	F (1, 23) = 0.07, p = .796, η^2_p = .00		
Alpha ERS	**F (1, 16) = 6.28, p = .023, η^2_p = .28**	F (1, 16) = 0.11, p = .746, η^2_p = .01		
Beta ERS	**F (1, 21) = 6.80, p = .020, η^2_p = .23**	F (1, 21) = 0.05, p = .831, η^2_p = .00		
fNIRS				
ΔHbO_2 peak	F (1, 36) = 0.05, p = .823, η^2_p = .00	F (1, 36) = 0.77, p = .387, η^2_p = .02		

A. Effects of Group (Old healthy vs Stroke), Hemisphere (Contralateral vs Ipsilateral) and Hand (Dominant / Non-paretic vs Non-dominant / Paretic) on the fNIRS and fEEG parameters on the paced-reaching task and **B.** Two levels interactions of the model. In bold, results with $p < .05$ and $\eta^2_p > .0$
